# Transitions in sense of coherence among middle-aged women raising adolescents before and during the COVID-19 pandemic

**DOI:** 10.3389/fpsyg.2023.1215463

**Published:** 2023-11-20

**Authors:** Tomoko Omiya, Tomoko Sankai, Akari Miyazaki, Yoshiki Abe, Naoko Kumada Deguchi

**Affiliations:** ^1^Division on Health Innovation and Nursing, Faculty of Medicine, University of Tsukuba, Tsukuba, Japan; ^2^Health Promotion Division of Ibaraki Prefectural Government, Mito, Japan; ^3^Faculty of Education, Shizuoka University, Shizuoka, Japan

**Keywords:** adolescents, COVID-19, job security, longitudinal study, middle-aged women, pandemic, sense of coherence, social capital

## Abstract

**Objective:**

A longitudinal study was conducted among Japanese women raising adolescents to determine how the COVID-19 pandemic has affected their sense of coherence (SOC) and to provide suggestions for supporting them.

**Methods:**

The SOC scores of 138 pairs of middle-aged women and their children (junior high school students) were compared at two time points: 2019 (before the pandemic, T1) and 2020 (during the pandemic, T2).

**Results:**

Overall, the women’s SOC did not change, whereas the adolescents’ SOC increased. In contrast, 44% of the women’s SOC scores decreased during the pandemic; no differences were detected between the SOC maintenance and increase group (G1) and the SOC decrease group (G2) in mental health, subjective physical health, social capital, and job status, and the child variables were not related. Multiple regression analysis of G1 and G2 with SOC at T2 as the dependent variable showed that for G2, at T1, having good mental and physical health conditions, a rich social capital, and having a job were positively associated with SOC during the pandemic.

**Conclusion:**

Middle-aged Japanese women, who often work as informal workers, are easily laid off and are involved in care work. Thus, the change in their socioeconomic status due to the pandemic may have been severe. Given the long-term social impact of the pandemic, it is necessary to consider support for women, including economic and social support such as employment and building human connections.

## 1 Introduction

The novel coronavirus disease (COVID-19) has caused serious stress in Japanese society. Following the declaration of a state of emergency on 7 April 2020, the government imposed various restrictions on economic activities and human flow. People drastically restricted their interactions with intimate ones in the name of social distancing and even changed their behaviour and social norms (Sugawara et al., 2022). The pandemic has also exacerbated health risk factors and chronic loneliness and has had various effects on individuals (Kikuchi et al., 2020; Lal et al., 2022).

As COVID-19 is an infectious disease, healthcare, food services, and hospitality industries, where interpersonal work is a mainstay, have been forced to cease or downsize their operations. In Japan, these jobs are primarily held by women as non-regular employees. Therefore, it is possible that women were most directly affected socially by the COVID-19 pandemic. Sakuragi et al. (2022) found that during the pandemic, the time women spent on housework and childcare increased compared to that of men. They pointed out that this increase may be related to the fact that women who worked informally lost their jobs due to the pandemic and that their husbands and children became homebound. In terms of mental health as well, Khan et al. (2022) indicated that during the pandemic, women were lonelier than men, especially middle-aged women, and older men were the loneliest. Koda et al. (2022) conducted a detailed data analysis on approximately 21,000 people reported in national suicide statistics from January 2020, when the first COVID-19 cases were identified in Japan, to May 2021. The analysis demonstrated that cases had increased, especially among women. The reasons were family problems, such as “child-rearing problems,” “marital discord,” and “fatigue from nursing care.”

In Japan, schools were closed nationwide in March 2020 because of COVID-19. Children were suddenly indoors 24 h a day for nearly 3 months. Moreover, COVID-19 made it difficult for people to receive medical and welfare services. Koda et al. (2022) suggested that fixed gender roles within Japanese society, where mothers assume childcare and caregiving roles, may contribute to the COVID-19 pandemic-related distress for women because of changes in their lives. The World Economic Forum (2022) also noted that the cost-of-living crisis had an unduly large impact on women, given the loss of the labour market due to the pandemic and the ongoing lack of care infrastructure. Owing to the pandemic, it is expected that many women and children will remain in poor economic, physical, and mental conditions. Therefore, support for them is an urgent priority.

Regarding the impact of the pandemic on children, a study examined how the “zest for life” of junior high school students attending a public school changed before and during the pandemic. Omiya et al. (2021) used sense of coherence (SOC) as an indicator of “zest for life” and conducted longitudinal assessments with 150 students in two schools to examine changes before and during the pandemic. The results demonstrated that although the overall SOC increased after the pandemic occurred, approximately 40% of the students continued to have a low SOC and displayed a lack of vitality or other such negative outcomes. It is possible that a certain number of adolescent children as well as middle-aged and older mothers of adolescents had lower SOC after pandemic onset.

Sense of coherence as a stress-coping skill is a core concept of the health generation theory proposed by health sociologist Antonovsky (1979, 1987). It is the ability to face stressful situations and circumstances and not only cope with them successfully but even transform them into sources of growth. SOC comprises three subconcepts: comprehensibility (confidence that predictions and explanations are possible), manageability (confidence that coping resources are always available), and meaningfulness (confidence that what happens is worth engaging with) (Yamazaki and Togari, 2011). Previous studies have demonstrated that SOC changes with life events in middle-aged and older women. The life course diverges in various ways after life events such as marriage, pregnancy, and childbirth. Yamazaki and Togari (2017) found no variation in SOC scores among middle-aged women regarding life course characteristics (e.g., single or married, housewife or working, and with or without children) and that SOC scores were relatively stable in mature age.

However, the pandemic seems to have had a negative impact on the SOC of Japanese women, who are considered the most vulnerable due to their precarious social status. In addition, women’s middle age coincides exactly with the adolescent child-rearing period, and this period also just corresponds to menopause, which is a time when mental and physical health problems are more likely to occur (Adachi, 2021). According to the Cabinet Office (2015), the middle-aged group comprises those aged 35–64 years, however, there is no consensus regarding the age range of those who are considered middle-aged. Further, middle-aged women are often in a difficult position as an increasing number of them are non-regular employees, they are in a precarious social position, and have limited opportunities for career advancement. The pandemic exacerbated this situation and further burdened these women. Such significant social changes have likely brought about changes in their SOC (Antonovsky, 1996). In particular, as with children, it is necessary to clarify the characteristics of the groups whose SOC has declined, to help improve the support for these populations during the ongoing pandemic.

This study aimed to capture changes in coherent sensory SOC before and during the pandemic among adolescents and middle-aged women who are raising the adolescents, and to identify the differences in and factors related to SOC, especially between the groups whose SOC decreased and those whose SOC increased. Additionally, this study aimed to present implications for providing support for middle-aged women during and after the COVID-19 pandemic.

## 2 Materials and methods

### 2.1 Participants

The sample comprised 138 students from two public junior high schools (A and B junior high schools) around the Tokyo area and their parents (mothers) who agreed to participate in the study. The baseline survey was conducted in the spring (March–April) of 2019 (before the pandemic; Time 1: T1), and the second survey was conducted from July to September of 2020 (Time 2: T2), after Japan declared a state of emergency due to the pandemic (declared at the end of February 2020). Most schools in Japan were closed for approximately 3 months, after which, students began attending school. Identification numbers (IDs) were matched between parents and students (children), and pairs whose data matched twice were included in the analysis.

### 2.2 Ethical considerations

As junior high students are minors, the survey was first explained in writing to their parents. Only those students who signed a consent form after obtaining handwritten consent from their parents were given the survey. Students were informed in writing, and their willingness to participate was confirmed by setting up a checkbox to indicate whether they agreed to participate in the survey. We also explained that participation in the survey was voluntary, that not participating in the study would not put them at a disadvantage, and that the participants are free to withdraw at any time. As this was a longitudinal study, each student was assigned a random six-digit personal ID number to manage the participant data. For parent–child data matching, a common parent–child number was assigned to each pair in addition to the individual ID number. However, these ID numbers were not personally identifiable, and anonymity was maintained throughout the study. Documents linking the personal ID number to the student’s name were maintained by one person at each school and locked in an archive so that individuals could not be identified based on their responses. Questionnaires were distributed to the students, which they answered. The students took the parent questionnaire home, for their parents to complete; the completed parent questionnaires were enclosed in sealed envelopes and delivered to the schools by the students. This study was conducted in accordance with the principles of the Declaration of Helsinki. This study was approved by the Medical Ethics Review Board of the University of Tsukuba (approval number: 1,343, approval date: 30 January 2019).

In a previous study (Omiya et al., 2021), we analysed the data from the child’s perspective. Conversely, in the current study, the data are analysed mainly from the parent’s perspective. However, the data for both studies were obtained simultaneously and analysed from different perspectives.

### 2.3 Methods

#### 2.3.1 SOC, a variable common to mother and child

The shortened Japanese version of the SOC scale, containing 13 items rated on a five-point scale, was used to measure SOC – a variable that is common to both mothers and children. Its reliability and validity have been verified by Togari and Yamazaki (2005). The scale has been widely used in Japanese national sample surveys and studies pertaining to high school students (Togari et al., 2012; Omiya et al., 2020, 2022). The SOC scale consists of three subscales: comprehensibility, manageability, and meaningfulness. The SOC scores were calculated for both parents and children at T1 and T2. The Cronbach’s alpha coefficients for the SOC ranged from 0.82–0.87 and 0.78–0.89 for mothers and children, respectively.

#### 2.3.2 Mothers

Regarding the variables examined at T1 and T2, mothers were asked about their age and marital status (married/unmarried, separated, or bereaved) as basic attributes. Previous research has demonstrated that SOC is associated with economic and relational wealth in the community (Koda et al., 2022), so we asked whether participants had a job at T1 (yes or no).

#### 2.3.3 Mental health inventory

This scale was used to measure mental health at T1 and T2. Yamazaki et al. (2005) translated the mental health inventory (MHI) developed by Berwick et al. (1991) into Japanese and validated it for use in Japanese populations. It is a 5-item tool (ranging from “always” to “never”) used to assess mental health problems, particularly depression, in the past month. Examples of items include “I was quite nervous” and “I was depressed and couldn’t help it.” Higher scores indicate better mental health. Cronbach’s alphas were 0.84 and 0.82 for both T1 and T2.

#### 2.3.4 Subjective physical health

We evaluated the participants’ subjective physical health by asking them questions regarding eight physical complaints (headache, stomach ache, insomnia, palpitations, vertigo, constipation/diarrhoea, body pain, and irritability), as suggested by Ben-Sira (1982). Measurements were taken at T1 and T2. The responses ranged from 1 “always” to 4 “never.” The higher the score, the better their subjective physical health. Cronbach’s alpha was 0.81 on this scale.

#### 2.3.5 Variable assessed only at T1: social capital

In a comparative study of SOC across regions, Tsuno and Yamazaki (2012) pointed out that reciprocal human relationships in the community are important factors for SOC. Especially during childcare, mothers’ perceptions of residents’ interactions with each other, such as rich human interactions and safe neighbourhoods, may be deeply related to SOC formation and change. Mothers’ perception of their neighbourhood was evaluated using Togari et al.’s (2015) scale, which comprises 6 items rated on a 5-point scale. Examples of the items include “The area where I live is very safe” and “We help each other.” The scale’s Cronbach’s alpha coefficient was 0.80.

#### 2.3.6 Measurement items for adolescents at T2

Adolescence has been described as a period of “storm and stress” (Hall, 1907), with the storm implying a decreased level of self-control and stress implying increased sensitivity at this stage of life. Considering the impact of the COVID-19 outbreak on this phase of life, we felt it necessary to examine the stress of children and its impact on their families (parents) during the pandemic. In this study, stress related to academic performance, friendship stress and family relationship stress was measured at T2 using the “Life-Related Stress Scale for High School Students,” which was developed by Ishida et al. (2017) and tested for reliability and validity. For academic performance stress, a 5-item, four-point scale was used, with higher scores indicating higher levels of stress about studying and grades. Friendship stress was assessed using a 5-item, four-point scale, and family relationship stress was assessed using a 4-item, four-point scale. Cronbach’s alpha coefficients were 0.88, 0.91, and 0.84, respectively.

### 2.4 Analysis

The sum of SOC scores at T1 and T2 were calculated for the 138 eligible mothers and their children. For the mothers, total scores at T1 and T2 were also calculated for the mental health inventory and subjective physical health. Corresponding *t*-tests were performed to determine differences in scores at the two-time points.

Next, based on previous studies (Omiya et al., 2021), we divided the mothers into two groups. Mothers were classified based on their total SOC score at T2 minus their total SOC score at T1 (T2-T1). If the result of the subtraction was zero or positive, the group was classified as “Group 1” (G1), the SOC maintenance and increase group, and if the result was negative, the group was classified as “Group 2” (G2), the SOC decrease group.

As a comparison between G1 (56.0%) and G2 (44.0%), *t*-tests and cross-tabulated χ^2^ tests were performed for baseline age and marriage as maternal variables. *t*-Tests were conducted for continuous variables and χ^2^ tests for all other variables. The same was conducted for social capital, where only T1 was measured. For children, *t*-tests were also conducted for T1 and T2 SOC total scores and for academic, friends, and family stress scores.

Pearson’s correlation coefficients were calculated for G1 and G2, mainly to establish the strength of association of the parent–child SOC score in T2 in a cross-sectional manner. Correlation coefficients with the parent’s T2 SOC total score and the child’s T2 SOC total score were calculated for the parent and child variables measured at T2. For social capital, correlation coefficients were calculated in the same way to examine inputs to the multiple regression analysis.

To explore the variables that influence the T2 maternal SOC score, multiple regression analysis was performed for each of the G1 and G2 groups using the forced entry method, with the T1 maternal variable as the explanatory variable and the T2 maternal SOC total score as the dependent variable. Based on the results of the analysis of the variables up to this point, we noted that the child variable was not well related to the mother’s SOC; therefore, we used the mother-only T1 variable as the explanatory variable. The explanatory variables were MHI, subjective physical health, social capital, and job status (yes = 1, no = 1), with age and marital status as control variables. Absolute correlations between independent variables were rejected.

## 3 Results

The difference between T1 and T2 scores is shown in Table 1. The results of the corresponding *t*-tests showed that the only variable that differed significantly between T1 and T2 was the child’s total SOC score. Children’s scores increased significantly from T1 to T2 (*p* = 0.002).

**TABLE 1:** Measured variables at T1 and T2 and comparison analysis results for each timeline.

Items	T1 2019, before pandemic occurred	T2 2020, during pandemic		Range, notes
	Average (SD)	Average (SD)	Paired *t*-test *p*-Value	
**Mothers**				
Sense of coherence (SOC)	42.6 (6.9)	42.8 (6.8)	0.687	Range: 13–65
Mental health inventory (MHI)	18.9 (3.1)	18.6 (3.0)	0.170	Range: 5–25, higher scores indicate good mental health
Subjective physical health	23.9 (4.4)	23.4 (4.1)	0.095	Range: 8–32, higher scores indicate good health
Employment status				
Employed, *n* (%)	110 (79.7%)	114 (82.6%)	–	
Unemployed, *n* (%)	28 (20.3%)	24 (17.4%)	–	
Social capital	21.1 (3.7)	–	–	Range: 6–30, higher is preferable
**Children**				
Sense of coherence (SOC)	40.9 (6.7)	42.8 (7.8)	0.002	Range: 13–65
Academic performance stress	–	9.2 (3.5)	–	Range: 5–20, higher scores indicate more experience
Friendship stress	–	5.7 (1.8)	–	Range: 5–20, higher scores indicate more experience
Family relationships stress	–	5.9 (2.7)	–	Range: 4–16, higher scores indicate more experience

Where the number of people did not match the total, it was a missing value.

Table 2 shows the comparative results of G1 and G2. *t*-Tests were conducted to compare groups, and no significant differences were detected in age, marital status, MHI, subjective physical health, or social capital for the mothers, except for the criterion SOC-related scores. For meaningfulness, a subscale of SOC, a score of 14.2 points was noted at T2, both for G1 and G2, and no significant difference was detected between the groups. For comprehensibility, scores of 16.8 for G1 and 15.5 for G2 were noted, with a significant *p*-value of 0.016 between the two groups at T2. For manageability, G1 scored 13.0 and G2 scored 11.5 at T2. The difference between the two groups was statistically significant at *p* < 0.001. No item was statistically significant (*p* < 0.05) between the two groups for the T2 child measurement variables.

**TABLE 2:** Score comparison results for each group.

Items	Group 1	Group 2	*t*-Test *p*-Value
	*n* = 77	*n* = 61	
	Average score (SD)		
**Mothers**			
Basic characteristics in baseline			
Age (SD)	45.5 (4.59)	44.6 (4.34)	0.217
Married	67 (87.0)	56 (91.8)	0.369 (χ^2^-test)
Never married, separated, or bereaved	10 (13.0)	5 (8.2)	
**T1 (2019)**			
Sense of coherence (SOC) total score	40.3 (6.5)	45.4 (6.4)	0.010
Meaningfulness	13.4 (2.4)	15.0 (2.5)	<0.001
Comprehensibility	15.5 (3.0)	17.2 (2.9)	<0.001
Manageability	11.3 (2.3)	13.3 (2.5)	<0.001
Mental health inventory (MHI) (range 5–25)	18.4 (3.3)	19.4 (3.2)	0.065
Subjective physical health (range 8–32)	23.6 (4.7)	24.4 (4.1)	0.280
Social capital (range 6–30)	20.9 (3.9)	21.2 (3.4)	0.570
Employed, *n* (%)	61 (79.2)	49 (80.3)	0.872 (χ^2^-test)
Unemployed, *n* (%)	16 (20.8)	12 (19.7)	
**T2 (2020)**			
Sense of coherence (SOC) total score	44.1 (6.9)	41.1 (6.3)	0.000
Meaningfulness	14.2 (2.3)	14.2 (2.4)	0.604
Comprehensibility	16.8 (3.4)	15.5 (3.1)	0.016
Manageability	13.0 (2.6)	11.5 (2.6)	<0.001
Mental health inventory (MHI) (range 5–25)	18.9 (2.7)	18.4 (3.1)	0.281
Subjective physical health (range 8–32)	23.8 (4.1)	23.1 (4.2)	0.314
Employed, *n* (%)	60 (77.9)	51 (83.6)	0.518 (χ^2^-test)
Unemployed, *n* (%)	17 (22.1)	10 (16.4)	
**Children (students)**			
SOC total score, T1 (range: 13–65)	40.4 (6.3)	42.0 (7.1)	0.198
SOC total score, T2 (range: 13–65)	42.6 (7.9)	44.3 (7.5)	0.215
Academic performance stress, T2 (range: 5–20)	9.3 (3.6)	8.2 (3.4)	0.069
Friendship stress, T2 (range: 5–20)	5.7 (1.9)	5.4 (0.9)	0.234
Family relationships stress, T2 (range: 4–16)	6.4 (3.1)	5.8 (2.4)	0.244

G1 is the mothers’ SOC increase/maintain group and G2 is the mothers’ SOC decrease group.

The results of the correlation analysis of variables associated with the SOC of T2 are shown in Table 3. Two variables were significantly correlated with the mother’s total SOC score at T2 for G1: MHI (*r* = 0.571, *p* < 0.01) and subjective physical health (*r* = 0.432, *p* < 0.01). For G2, MHI (*r* = 0.641, *p* < 0.01) and subjective physical health (*r* = 0.488, *p* < 0.01) as well as social capital (*r* = 0.330, *p* < 0.01) showed a significant positive correlation. None of the child variables were significantly correlated. Correlation coefficients for children’s SOC scores at T2 also showed no significant correlation with mothers’ measured scores in both groups, and except for G2 friendship stress, children’s T2 SOC was negatively associated only with children’s own stress-related variables (*r* = −0.563 to −0.293, *p* < 0.05 to 0.01).

**TABLE 3:** Correlation coefficient with SOC total score of the two groups.

	Group 1		Group 2	
Measurement items at T2	*n* = 77		*n* = 61	
	Correlation coefficient (*r*)		Correlation coefficient (*r*)	
	With mothers’ SOC total score (T2)	With children’s SOC total score (T2)	With mothers’ SOC total score (T2)	With children’s SOC total score (T2)
**Mothers’**				
SOC score in 2020	–	0.036	–	0.170
Mental health inventory (MHI)	0.571**	0.063	0.641**	0.230
Subjective physical health	0.432**	0.080	0.488**	0.250
Social capital, data in T1	0.205	−0.026	0.330**	−0.021
**Children’s**				
SOC score in 2020	0.036	–	0.170	–
Academic performance stress	−0.059	−0.563**	−0.042	−0.367**
Friendship stress	−0.125	−0.293*	0.050	−0.240
Family relationships stress	−0.038	−0.437**	0.165	−0.325*

G1 is the mothers’ SOC increase/maintain group and G2 is the mothers’ SOC decrease group. *p < 0.05, **p < 0.01.

For G1 and G2, multiple regression analysis was performed with T2’s total maternal SOC score as the dependent variable. The explanatory variables were the measured variables at T1, and we aimed to examine the effect of T1 on T2 SOC. Table 4 presents the results. For G1, the significant items were MHI (β = 0.379, *p* = 0.001) and subjective physical health (β = 0.301, *p* = 0.005). The total adjusted *R*^2^ was 0.378.

**TABLE 4:** Factors related to mothers’ SOC (T2) of the two groups.

Mothers’ variables in T1	Group 1 (*n* = 77)						Group 2 (*n* = 61)					
	*T* score	Coefficient β	*p*-Value	Standard error	95% CI	VIF	*T* score	Coefficient β	*p*-Value	Standard error	95% CI	VIF
Age	2.022	0.204	0.047	0.175	0.005 to 0.703	1.094	1.515	0.162	0.136	0.151	−0.074 to 0.533	1.210
Marital status (married = 1, others = 0)	−0.974	−0.098	0.333	2.229	−6.621 to 2.278	1.081	1.794	0.203	0.079	2.414	−0.515 to 9.177	1.354
Mental health inventory (MHI)	3.447	0.379	0.001	0.239	0.347 to 1.301	1.302	3.463	0.386	0.001	0.211	0.308 to 1.156	1.317
Subjective physical health	2.888	0.301	0.005	0.179	0.159 to 0.873	1.171	3.686	0.380	0.001	0.154	0.258 to 0.876	1.128
Social capital	0.350	0.036	0.728	0.186	−0.306 to 0.436	1.161	2.866	0.300	0.006	0.183	0.157 to 0.892	1.163
Employment status (employed = 1, unemployed = 0)	−0.854	−0.085	0.396	1.661	−4.735 to 1.897	1.055	2.046	0.213	0.046	1.594	0.062 ot 6.461	1.152
*R*		0.615						0.721				
*R* ^2^		0.378						0.520				
Total adjusted *R*^2^		0.322, *p* < 0.001						0.463, *p* < 0.001				

G1 is the mothers’ SOC increase/maintain group and G2 is the mothers’ SOC decrease group. All explanatory variables (of mothers) were measured at T1. Age and marital status are control variables.

For G2, significant positive associations were found for MHI (β = 0.386, *p* = 0.001) and subjective physical health (β = 0.380, *p* = 0.001) as well as social capital (β = 0.300, *p* = 0.006) and having a job (β = 0.213, *p* = 0.046). The adjusted *R*^2^ was 0.463. No multicollinearity was observed in both groups based on VIF values.

## 4 Discussion

This study is considered noteworthy as it is the first, to the best of our knowledge, to examine adolescents’ and their mothers’ stress-coping skills before and during the COVID-19 outbreak.

### 4.1 Overall SOC score

Antonovsky, who proposed the core concept of SOC, stated that SOC is stable in adulthood and gradually increases with age. Thus, there was no change in SOC scores over the approximate one-year span of this study (Antonovsky and Sagy, 1986; Antonovsky, 1987, 1996). However, the increase in SOC for adolescents may have been due to the reopening of schools after the first emergency declaration issued in Japan and the ensuing simultaneous school closures. The fact that adolescents’ SOC scores increased regardless of the group to which their parents belonged suggests that school life significantly impacts adolescents. It can also be inferred that the SOC scores of the parents and their children were not significantly related.

Moreover, approximately 40% of the mothers’ scores decreased. Interestingly, of the three components of SOC, there was no decline in meaningfulness, suggesting that the score decline in G2 was due to the other two components: comprehensibility and manageability. According to Yamazaki and Togari (2011), comprehensibility is “the ability to predict, to some extent, the various events one encounters daily and explain what those events are like.” The literature on stress-coping skills suggests that the key to improving comprehensibility is to foster consistent experiences. As mentioned earlier, the COVID-19 pandemic has changed society, causing a shift in daily lifestyles. As it would have been for anyone, the pandemic would still have been difficult for women to predict or explain. The government issued a state of emergency declaration and a request to close schools in Japan. Three months later, they were lifted despite the ongoing COVID-19 pandemic. We surmise that this would not have been a confusing and inconsistent experience for women.

Manageability is “the confidence that one is capable of using the coping resources (people, goods, tools, and thinking skills) necessary to overcome the events of daily life.” Moreover, to enhance this, “a balanced load experience that is neither too much nor too little” is considered valuable (Yamazaki and Togari, 2011). For many mothers who manage family life, a situation where a child cannot attend school may have been too sudden and burdensome. It is also conceivable that they may have experienced a situation where they could not find any means of coping at all, such as the lack of coping resources – for example, facilities to take care of their children, cram schools, and neighbours – and they had to go to work. In Japan, many children disrupt their lifestyles during school vacations by turning their days and nights upside down or becoming immersed in their smartphones. If the cause or trigger were school closures due to the COVID-19 pandemic, parents might not have been able to find an effective solution. These experiences may have lowered manageability.

In Japan, under the Infection Control Act, the treatment for COVID-19 has changed since May 2023, and COVID-19-related restrictions are loosening (Infectious Disease Subcommittee of the Health Sciences Council, 2023). However, it is possible that the damage caused by the pandemic, which lasted for three years, negatively impacted women, a socially vulnerable group. These trends should be monitored in future studies.

### 4.2 Comparison between the two groups and multiple regression analysis results

The two groups were compared; however, no significant differences were detected. The authors asked the participants (mothers) about their employment status, considering the possibility of economic damage related to COVID-19. We expected these to have some relationship; however, we found no difference between the two groups. General anxiety about daily life, life changes, or a sense of self-efficacy due to the COVID-19 pandemic may have made a difference rather than something more direct, such as a specific event. The results in Tables 1–3 also suggest that for parents and children, the relationship between them would be very weak, even for SOC and other variables. It has been previously suggested that school life, in which adolescents spend most of their time, especially more than the parent–child relationship, is associated with SOC (Omiya et al., 2022). Thus, support for both the parent and child need to be considered.

Multiple regression analysis revealed that the significantly associated factors differed between the two groups. This section will mainly discuss the group with lower SOC (G2) to provide suggestions to support the women in the G2 condition. The MHI and subjective physical health scores pertain to the subjective health of mind and body, respectively, with higher scores indicating better mental and physical health. Many studies have reported strong positive associations or causal relationships between the variables related to the relationship between SOC and mental and physical health. As expected, the results were interesting: the coefficient β for social capital was lower than the coefficients β for MHI and subjective physical health, but the *p*-value scores were considered sufficient to be significant.

Several studies have pointed out the relationship between social capital and SOC (Larm et al., 2016; van Sint Fiet et al., 2022). SOC assumes a self that exists with people and the surrounding environment and attempts to measure the degree of trust in one’s environment and the people in one’s life. In other words, SOC has a high affinity for social mechanisms based on trust, norms, and networks, which are characteristics of social capital, as proposed by the political scientist Putnam (2000).

The reason social capital has a significant positive association only in G2 must be interpreted. Social capital refers to the relationship between people in the community, including cooperation and interaction, mutual support, and a sense of security. The relationship between the community and SOC has been pointed out in the past (Tsuno and Yamazaki, 2012); for example, it has been reported that building mutually beneficial relationships in the community is related to SOC (Fukuda and Fukuda, 2022). In G2, warm connection, security, and support in the community as social capital may have acted as a coping resource, even when the family was disrupted, or the child became unstable due to the effects of the COVID-19 pandemic. For people in G2, peace of mind and a sense of de-isolation due to the richness of social capital may have been a relief. It is also possible that the richness of social capital improved SOC through manageability.

For women in G2, employment was also positively and significantly associated with SOC scores; as noted earlier, the economic hardship caused by the COVID-19 pandemic was extremely damaging, especially for women working informally in Japan. Therefore, it is plausible that having a job and being able to earn money, regardless of whether they are employed full time or part time, would have led to a certain degree of economic security. The link between SOC and job availability has been mentioned previously (Volanen et al., 2004; Feldt et al., 2005). SOC may be associated not only with the value of money itself but also with the social value of one’s activities and a sense of contribution through work. It is also possible that the work may have strongly affected the participants’ sense of place in the context of reduced social interaction.

Thus, it is possible that the items that were significantly associated with SOC in G2 were tangible and intangible, providing important support for the difficulties that middle-aged women were likely to face, such as loneliness, helplessness, and economic insecurity caused by the COVID-19 pandemic. Therefore, it is possible that not only financial or tangible means of support but also the creation of relationships and networks that enable small interactions (i.e., small talk or chatting), as well as events and interventions that foster relationships, may provide women with the required support (Pasek et al., 2017; Nowicki et al., 2020).

### 4.3 Limitations

Although we targeted mothers with children attending junior high school and obtained cooperation from two junior high schools, the data are limited as few parent–child pairs were available at both time points. Therefore, caution is required when generalising the results. Although, regrettably, it is no longer possible to obtain matching data from before the pandemic, we believe it is necessary to accumulate research from a long-term perspective by designing longitudinal studies and conducting analyses related to SOC with a focus on middle-aged women. We are convinced that such a focus will have implications for the SOC theory, stress theory, midlife support, and support for menopausal women.

As presented in Table 2, there were no significant differences between the two groups. Although not included in the text or the tables, the participants were asked to answer 20 items regarding life events after the onset of the pandemic (e.g., divorce, need for nursing care, and company bankruptcy). There was no difference between the two groups in this regard. Differences may have been observed for some items not included in this study. However, on the three subscales of the SOC component, differences were found between the two groups regarding comprehensibility and manageability. For example, it is important to clarify which specific items related to these two subscales differed between the two groups in the interviews to provide clues for providing support. In addition, the participants were biased in that they were highly aware of and interested in the issues and agreed to participate in this type of study. Those who did not respond to the survey were more likely to experience problems; thus, further research is required.

## 5 Conclusion

In Japan, often, women are non-regular employees and engage in care work such as housework, childcare, and nursing care, placing them in a socially vulnerable position. We considered the possibility that the COVID-19 pandemic might have impacted women raising adolescent children. We followed changes in the SOC of 138 parent–child pairs using SOC scores as an indicator to clarify the impact of the pandemic. Overall, there was no change in the scores of middle-aged mothers before and during the pandemic, whereas the scores of their children (junior high school students) increased. However, 44% of mothers had lower SOC scores than before the pandemic, with particularly significant declines in comprehensibility and manageability scores among the SOC components. For example, anxiety about uncertain prospects may be associated with lower SOC; in the group with declining SOC, perceived high social capital and employment may have contributed to stable SOC. Conversely, the results suggest that among middle-aged women raising adolescents in the community, maintaining social interactions is much more important, such as living in a community where they can casually talk and communicate. Work is also important for economic security. Simultaneously, having places to spend their time outside the home setting, where they feel safe, is also important. Further research is required to investigate these long-term implications.

## Data availability statement

The datasets presented in this article are not readily available because it contains data on minors and we do not have permission to share the data from the middle school and parents who cooperated with the survey. Requests to access the datasets should be directed to TO, toomiya-tky@umin.ac.jp.

## Ethics statement

This study was conducted in accordance with the Declaration of Helsinki and approved by University of Tsukuba’s medical ethics review board approved this research (approval number: 1,343, approval date 30 January 2019: 1343-1, 10 June 2020). Informed consent was obtained from all the participants involved in the study. Upon the minors’ participation in the study, we obtained their parents’ handwritten signatures. The students (junior high school students) were then allowed to express their willingness to participate in the study on their own. The authors provided a written statement from the authors that they would not be disadvantaged in any way if they did not participate in the study or if they dropped out of the study.

## Author contributions

TO and ND contributed to the conception, methodology, coordination of the survey participants, and in the refinement of the questions to capture the characteristics of the target population. TO wrote the entire manuscript. AM and TS contributed to the discussions and provided important inputs regarding manuscript editing and conference presentations. YA provided important advice on variable adjustment and analysis and contributed to the presentation and interpretation of the results. All authors contributed to the manuscript revision, read, and approved the submitted version.
